# Andrographolide Suppresses MV4-11 Cell Proliferation through the Inhibition of FLT3 Signaling, Fatty Acid Synthesis and Cellular Iron Uptake

**DOI:** 10.3390/molecules22091444

**Published:** 2017-08-31

**Authors:** Xiao Chen, Jianbin Zhang, Lixia Yuan, Yifei Lay, Yin Kwan Wong, Teck Kwang Lim, Chye Sun Ong, Qingsong Lin, Jigang Wang, Zichun Hua

**Affiliations:** 1 The State Key Laboratory of Pharmaceutical Biotechnology, School of Life Sciences, Nanjing University, Nanjing 210023, China; pyb025@126.com; 2 Department of Oncology, Clinical Research Institute, Zhejiang Provincial People’s Hospital, People’s Hospital of Hangzhou Medical College, Hangzhou 310014, China; zhangjianbin@hmc.edu.cn; 3 School of Traditional Chinese Medicine, Southern Medical University, Guangzhou 510515, China; cnylxtcm@163.com; 4 Department of Biological Sciences, National University of Singapore, Singapore 117543, Singapore; layyifei@gmail.com (Y.L.); e0146526@u.nus.edu (Y.K.W.); dbslimtk@nus.edu.sg (T.K.L.); 5 Institute of Mental Health, Education Office, Singapore 539747, Singapore; Chye_Sun_ONG@imh.com.sg; 6 Changzhou High-Tech Research Institute of Nanjing University, Institute of Biotechnology, Jiangsu Industrial Technology Research Institute and Jiangsu Target Pharma Laboratories Inc., Changzhou 213164, China

**Keywords:** andrographolide, fatty acid synthesis, FLT3 signaling, intracellular iron pool, MV4-11, protein synthesis, quantitative proteomics

## Abstract

*Background*: Andrographolide (ADR), the main active component of *Andrographis paniculata*, displays anticancer activity in various cancer cell lines, among which leukemia cell lines exhibit the highest sensitivity to ADR. In particular, ADR was also reported to have reduced drug resistance in multidrug resistant cell lines. However, the mechanism of action (MOA) of ADR’s anticancer and anti-drug-resistance activities remain elusive. *Methods*: In this study, we used the MV4-11 cell line, a FLT3 positive acute myeloid leukemia (AML) cell line that displays multidrug resistance, as our experimental system. We first evaluated the effect of ADR on MV4-11 cell proliferation. Then, a quantitative proteomics approach was applied to identify differentially expressed proteins in ADR-treated MV4-11 cells. Finally, cellular processes and signal pathways affected by ADR in MV4-11 cell were predicted with proteomic analysis and validated with in vitro assays. *Results*: ADR inhibits MV4-11 cell proliferation in a dose- and time-dependent manner. With a proteomic approach, we discovered that ADR inhibited fatty acid synthesis, cellular iron uptake and FLT3 signaling pathway in MV4-11 cells. *Conclusions*: ADR inhibits MV4-11 cell proliferation through inhibition of fatty acid synthesis, iron uptake and protein synthesis. Furthermore, ADR reduces drug resistance by blocking FLT3 signaling.

## 1. Introduction

Acute myeloid leukemia (AML) is characterized by the uncontrolled proliferation of immature hematopoietic precursors with reduced potential to differentiate into their downstream myeloid counterparts [1,2]. Compared to other common types of leukemia, AML attracts considerable attention due to its relatively high incidence rate of about 3.6 per 100,000 person, around two-fold that of acute lymphocytic leukemia and chronic myeloid leukemia [3]. The survival prospect of AML patients is also one of the worst, with less than 40% patients surviving longer than 12 months after diagnosis [4]. Among less tolerant elderly patients, serious complications often arise after aggressive treatments. Treatment failure in patients of all ages, however, are mainly caused by the increasing resistance towards therapy, with 50% of patients achieving complete remission experiencing relapse [5,6].

The receptor tyrosine kinase FLT3-ITD is known as one of the major molecular genetic abnormalities which confers chemotherapy drug resistance and high relapse risk among 23% of AML patients [7]. It is expressed in early hematopoietic progenitor cells and activates downstream PI3K/Akt and RAS/MAPK pathways governing early stem cell survival and myeloid differentiation [8,9]. A few studies have reported an association of the FLT3-ITD mutation with a poor prognosis- specifically, low complete remission rate and high relapse risk as well as adverse disease-free survival and overall survival [10,11]. Although several FLT3 tyrosine kinase inhibitors have been developed for exclusive treatment of ITD positive patients, the effects are rather transient and drug resistance developed shortly after treatment [12,13].

Conscious efforts for anti-cancer therapeutic compounds from plant sources have been made since the 1950s, leading to the discovery of several well-known chemotherapeutic drugs such as vinblastine [14], podophyllotoxins [15], the camptothecins [16], and artemisinin [17,18]. In recent years, phytochemicals found in dietary and medicinal plants in the tropical and sub-tropical regions are frequently tried for their potential anti-neoplastic properties [19,20]. Andrographolide (ADR), a labdane diterpene isolated from the leaves of *Andrographis paniculata* Nees, is one such phytochemical which exhibits an extraordinarily wide range of biological activities [21,22,23,24]. Since its first discovery by Gorter in 1911, ADR has been reported to have potent cytotoxicity effects on various types of cancer [25,26,27]. In particular, ADR also exhibits anticancer activity against AML cells. For instance, ADR was discovered to induce cell cycle arrest at G0/G1 phase in AML HL-60 cells by modulating the expression of cell cycle proteins [28]. Furthermore, in a screening of 60 cancer cell lines consisting of nine cancer types, leukemia cell lines (CCRF-CEM, K562, MOLT-4, RPMI-8226 and SR) on average showed the highest sensitivity to ADR in comparison to CNS, colon, ovarian, renal, prostate and breast cancer cell lines [26].

A large number of studies have proved that ADR is involved in various cellular processes in a variety of cancer cell lines, such as cell cycle [29], cell apoptosis [30], cell proliferation [31], inflammation [21] and angiogenesis [32]. However, the specific mechanism of action of ADR on AML cells remains elusive. Moreover, as ADR was reported to exert efficient cytotoxicity against HCT-8/5FU multidrug resistant colorectal cancer cell line [33], we hypothesized that ADR also displays therapeutic effect on multidrug resistant AML cell line. Therefore, MV4-11 AML cancer cell line, a FLT3-positive cell line which exhibits multidrug resistance [34], was included in our experimental system to investigate the mechanism of ADR’s anticancer activity, especially ADR’s effect on multidrug resistant cancer cells.

In the present study, we first evaluated the effect of ADR on MV4-11 cell proliferation. Next, a quantitative proteomics approach was applied to identify differentially expressed proteins in MV4-11 cells with ADR treatment. With proteomic analysis, we predicted the ADR-modulated cellular processes and signaling pathways and elucidated the mechanism of action of ADR against multidrug resistant AML cancer cells.

## 2. Results

### 2.1. ADR Inhibits MV4-11 Cell Proliferation in a Dose- and Time-Dependent Manner

**Figure 1:**
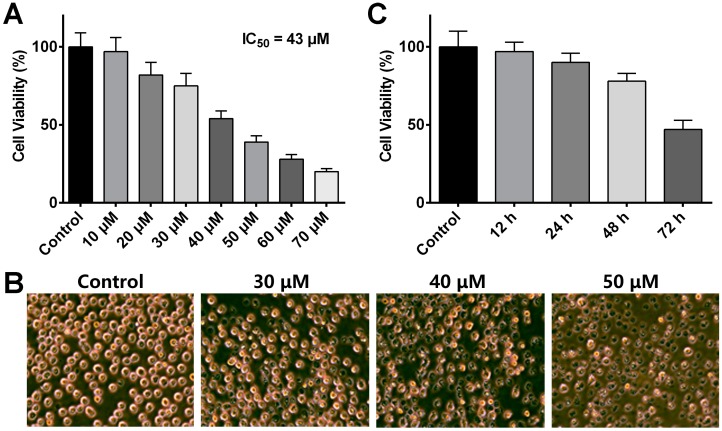
(**A**) Cell viability of MV4-11 cells treated with different concentrations of ADR; (**B**) Microscopic images of the cell culture treated with different concentrations of ADR; (**C**) Cell viability of MV4-11 cells treated with 43 μM ADR for different time span.

To determine the effect of ADR on MV41-11 cell proliferation, a CCK-8 assay was conducted to detect the cell viability of MV4-11 cells treated with different concentrations of ADR for 72 h. Results showed that ADR inhibited MV4-11 cells proliferation in a concentration-dependent manner, and the IC_50_ value of ADR was 43 μM (Figure 1A). Microscopic images of the cell cultures were also taken to observe the morphological changes of the cells upon 72 h of treatment with various concentrations of ADR (Figure 1B). In the same fashion, almost 50% of the cell population turned flaccid and dark coloured at the dosage level of 40 μM, indicating the big loss of cell viability. Next, we exposed MV4-11 cells to 43 μM ADR for different time span ranging from 0 h to 72 h, and results showed that ADR inhibits cell proliferation in a time-dependent manner (Figure 1C). As ADR treatments for 24 h and 48 h exhibit mild cytotoxicity against MV4-11 cells, we adopt 43 μM as our experimental concentration and 72 h as drug administration time in subsequent assays. List of top 100 overexpressed proteins and underexpressed proteins at 72 h post-ADR treatment is available at Tables S1 and S2.

### 2.2. Using Quantitative Proteomics Approach to Identify Differentially Expressed Proteins in ADR-Treated MV4-11 Cells

**Figure 2:**
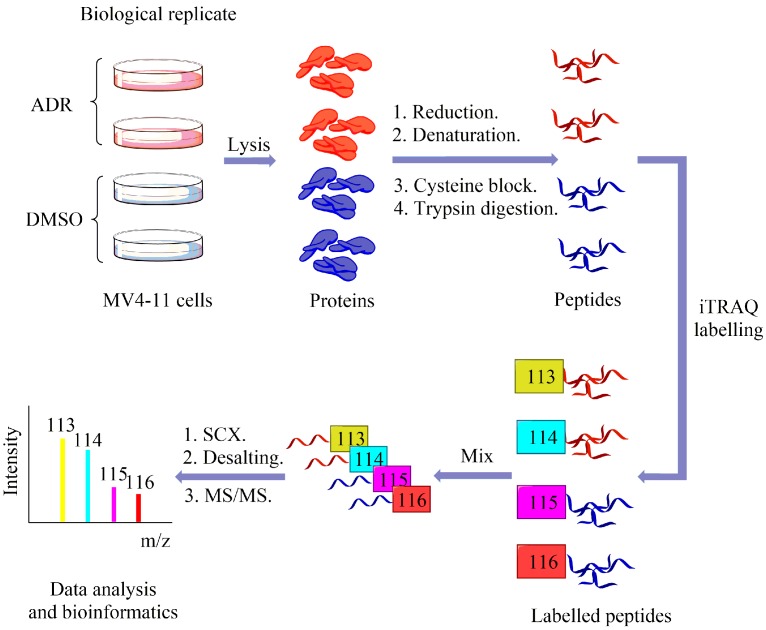
General workflow of iTRAQ coupled with LC-MS/MS.

**Figure 3:**
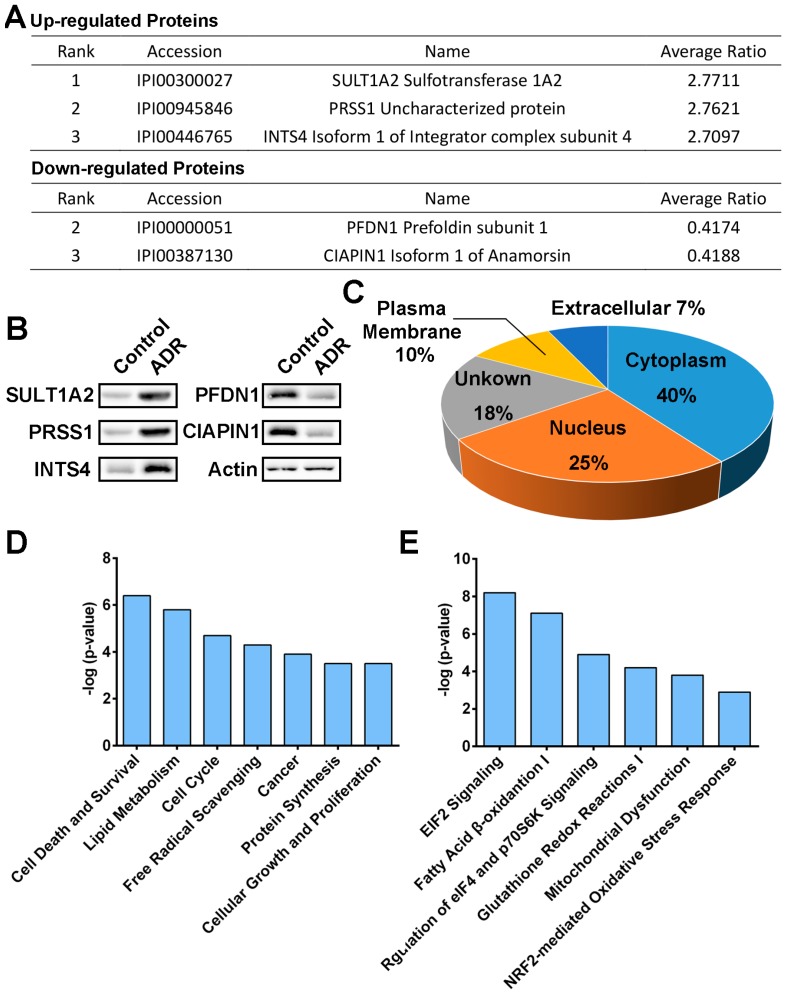
(**A**) Five of the most ADR-regulated proteins; (**B**) western blotting validation of the five proteins; (**C**) cellular distribution of ADR-modulated proteins in MV4-11 cells; (**D**) ADR-regulated cellular functions in MV4-11 cells from IPA analysis; (**E**) ADR-regulated pathways in MV4-11 cells from IPA analysis.

To elucidate the mechanism of ADR action against MV4-11 cells, an iTRAQ approach, coupled with LC-MS/MS, was applied to identify significantly modulated proteins in MV4-11 cells with 43 μM ADR treatments for 72 h (Figure 2). Briefly, MV4-11 cells were treated with ADR or DMSO (control) for 72 h in parallel (two replications for each treatment), followed by cell lysis, reduction, denaturation, cysteine blocking and trypsin digestion. Then, the peptides of each group were labelled with their respective iTRAQ reagents (ADR-treated samples were labeled with 113 or 114, while control samples were labeled with 115 or 116. 113, 114, 115 or 116 refers to the mass of the reporter group in iTRAQ reagents). After iTRAQ labelling, all the peptides were pooled together, purified with cation exchange column, desalinated with desalting column and analyzed with LC-MS/MS to identify differentially expressed proteins. The average iTRAQ ratio is the average of 113/115, 114/115, 113/116 and 114/116. A strict cutoff threshold (*p*-value < 0.05 and the average iTRAQ ratio for upregulated proteins > 1.26 while for down regulated proteins < 0.78) was employed to guarantee the specificity of the results. 522 proteins were picked out from a total of 3604 identified proteins to be significantly regulated by ADR. List of top 100 overexpressed proteins and underexpressed proteins at 72 h post-ADR treatment is available at Tables S1 and S2. To further validate the identified proteins as the ADR-modulated proteins, three of the most overexpressed proteins (SULT1A2, PRSS1 and INTS4) and two of the most underexpressed proteins (PFDN1 and CIAPIN1) were subjected to immunoblotting with their respective antibodies (Figure 3A,B). Results emphatically confirmed the regulating effect of ADR on the proteins.

The cellular distribution of the significantly modulated proteins was analyzed with the aid of DAVID gene ontology database. Around two-third of the proteins were characteristically localized in the cytoplasm and the nucleus (Figure 3C), suggesting that ADR initiates its anticancer activity by affecting cytosolic and nuclear protein functions. Ingenuity Pathway Analysis (IPA) was used to identify ADR-modulated cellular functions and pathways. As shown in Figure 3D, ADR affected various cellular functions in MV4-11 cells, including cell death and survival, free radical scavenging, cell cycle, lipid metabolism, cancer, protein synthesis and cellular growth and proliferation, indicating the cytotoxic effect of ADR on cancer cells. In addition, the top five ADR regulated pathways included EIF2 signaling, fatty acid β-oxidation I, regulation of eIF4 and p70S6K signaling, glutathione redox reactions I and mitochondrial dysfunction (Figure 3E).

### 2.3. ADR Inhibits MV4-11 Proliferation Through Suppressing Fatty Acid Synthesis

**Figure 4:**
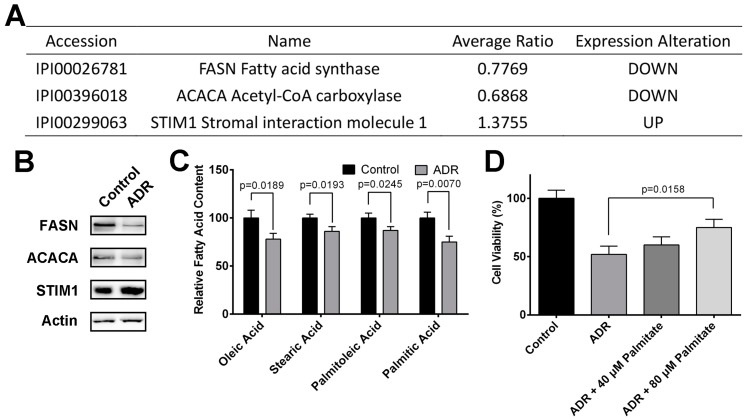
(**A**) ADR modulated proteins involved in fatty acid synthesis; (**B**) western blotting validation of the proteins; (**C**) the effect of ADR on several fatty acid contents in MV4-11 cells; (**D**) the effect of different concentrations of palmitate on ADR-treated MV4-11 cells viability.

Alteration of lipid metabolism has been increasingly recognized as the prominent feature of cancer pathogenesis [35]. Continuous lipogenesis provides cancer cells with membrane building blocks, signaling lipid molecules, posttranslational modifications of proteins as well as energy supply to support rapid cell proliferation [36]. According to IPA results, ADR might affect lipid metabolism and fatty acid β-oxidation I pathway in MV4-11 cells. Figure 4A summarizes the ADR-regulated proteins that are involved in lipid metabolism and fatty acid synthesis, including FASN, ACACA and STIM1, and the modulation effect of ADR on their expression were validated by western blotting (Figure 4B). FASN is a multienzyme complex which catalyzes the formation of saturated fatty acid palmitate from NADPH-dependent condensation of acetyl-CoA and malonyl-CoA [37]. ACACA, as a complex multifunctional enzyme system, is essential for fatty acid synthesis [38]. STIM1 is an activator of AMPK pathway closely relates to FASN and ACACA expression [39]. Our results showed that ADR down-regulated FASN and ACACA expression, while up-regulated STIM1, suggesting that ADR inhibits fatty acid synthesis in MV4-11 cells. To validate the conclusion, we utilized GC/MS analysis to determine the fatty acid content alteration in MV4-11 cells with ADR treatment. Consistently, ADR significantly decreased several fatty acid contents in MV4-11 cells, including oleic acid, stearic acid, palmitoleic acid and palmitic acid.

To find out whether ADR-induced inhibition of fatty acid synthesis contributes to its cytotoxicity on MV4-11 cells, we incubated ARD-treated MV4-11 cells with various concentration of palmitate-BSA complex. The results suggested that palmitate-BSA rescued MV4-11 cells from ADR’s cytotoxicity in a concentration-dependent manner to some extent (Figure 4D), indicating that the inhibitory effect of ADR on fatty acid synthesis at least partially contributes to the cytotoxicity of ADR.

### 2.4. ADR Suppresses Iron Uptake and Induces Retention of Iron in Storage Repositories to INHIBIT MV4-11 Cells Proliferation

**Figure 5:**
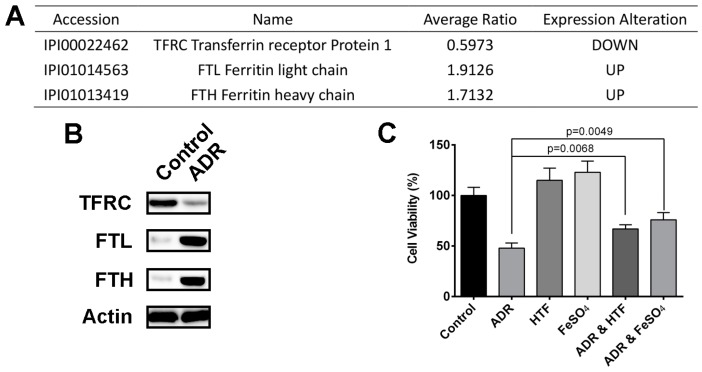
(**A**) ADR modulated proteins involved in intracellular iron regulation; (**B**) western blotting validation of the proteins; (**C**) the effect of HTF and FeSO_4_ on ADR-treated MV4-11 cells viability.

Iron is an essential element required for various vital physiological functions ranging from gas transportation to energy metabolism to DNA synthesis [40]. Compared to normal cells, tumor cells always display enhanced iron absorption and cellular uptake in order to drive rapid growth and proliferation [41]. Previous reports have correlated increased intracellular iron deposition with enhanced cell proliferation in a variety of cancers, including colorectal cancer, breast cancer, adenocarcinomas, chronic lymphocytic leukemia and AML. In the present study, three proteins, TFRC, FTL and FTH, in our ADR-modulated protein list are involved in intracellular iron regulation (Figure 5A). Proteomic data showed that ADR down-regulated TFRC while up-regulated FTL and FTH. Western blotting was conducted to validate the regulation effect of ADR on the expression of these proteins (Figure 5B).

TFRC1, a membrane glycoprotein, mediates cellular iron uptake by undergoing endocytosis upon binding of iron-loaded transferrin (TF) [42]. In the acidified endosomes, the ferric iron is released from the TF-TFRC complex and subsequently transported to the cytosol for cellular utilization. Ferritin (FT) is a ubiquitous intracellular protein, which assembles into a hollow cage-like structure for storage of excess Fe^3+^ ions in the form of ferric oxyhydroxide phosphate (see Figure 8). Therefore, we concluded that ADR decreases intracellular iron pool in MV4-11 cells through inhibition of iron uptake and retention of iron in storage repositories. To further investigate the relationship between decreased intracellular iron pool and cell proliferation inhibition, we utilized holotransferrin (HTF) and iron sulphate (FeSO_4_) as exogenous iron source to treat ADR-treated MV4-11 cells. Results showed that HTF and FeSO_4_ rescued MV4-11 cells from ADR’s cytotoxicity to some extent (Figure 5C), indicating ADR inhibits MV4-11 cell proliferation through decreasing intracellular iron pool.

### 2.5. ADR Blocks FLT3 Signaling Pathway and Suppresses Protein Synthesis in MV4-11 Cells

**Figure 6:**
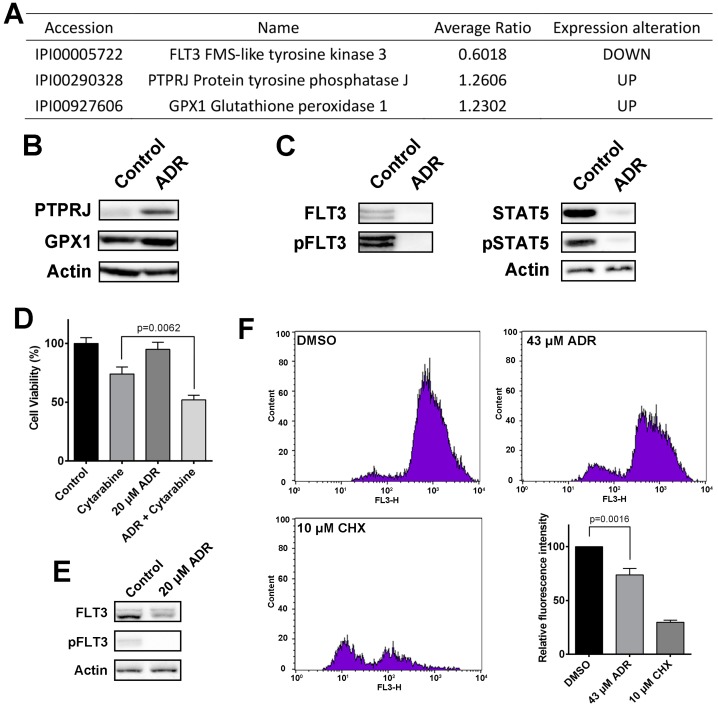
(**A**) ADR modulated proteins involved in FLT3 pathway; (**B**) western blotting validation of the proteins; (**C**) the effect of ADR on the expression of key proteins in FLT3 pathway; (**D**) cell viability of MV4-11 cells treated with 2 μM cytarabine for 24 h, 20 μM ADR for 24 h, or 20 μM ADR for 24 h followed by 2 μM cytarabine for 24 h; (**E**) the effect of 20 μM ADR on FlT3 signal pathway in MV4-11 cells; (**F**) reduction of protein synthesis by ADR in MV4-11 cells.

As MV4-11 cell line is characterized by homozygous FLT3-ITD mutation and activated FLT3 drives multidrug resistance of the cell line, FLT3 signaling pathway in response to ADR treatment was considered the most important factor that influences ADR’s anticancer effect. Three ADR-modulated proteins were involved in FLT3 signaling pathway, including FLT3, PTPRJ and GPX1 (Figure 6A). Proteomic data showed that ADR down-regulated FLT3, while up-regulated PTPRJ and GPX1. Results from western blotting validated this conclusion (Figure 6B,C). PTPRJ was reported to negatively regulate FLT3 phosphorylation and downstream signaling activity, and depletion of PTPRJ was associated with site-selective hyperphosphorylation of FLT3 [43]. GPX is an antioxidant enzyme involved in catalyzing the decomposition of harmful H_2_O_2_, which reverses H_2_O_2_-induced inactivation of PTPRJ, thereby allowing PTPRJ to dephosphorylate FLT3 and inhibit its downstream signaling [44]. 

Taken together, we assume that ADR blocks FLT3 signaling pathway through up-regulating GPX and PTPRJ. Western blotting was conducted to determine the effect of ADR on FLT3 signal pathway, and ADR significantly decreased the expression of FLT3 and phosphorylated FLT3 (Figure 6C), suggesting that ADR inhibits FLT3 signal pathway. In addition, as FLT3 drives its downstream STAT5 activation, the inhibition of ADR on STAT5 signaling reinforced ADR’s inhibitory effect on FLT3 signal pathway (Figure 6C). Considering activated FLT3 signaling drives multidrug resistance in MV4-11 cells, we introduced a chemotherapy drug, cytarabine, to examine the effect of ADR on the drug resistance of MV4-11 cells [45]. Treatment with cytarabine alone resulted in a limited therapeutic effect (Figure 6D), revealing the drug resistance of MV4-11 cells. As shown in Figure 6D, treatment with 20 μM ADR for 24 h exhibited weak inhibitory effect on MV4-11 cells, but still significantly suppressed FLT3 signaling in MV4-11 cells (Figure 6E). In addition, pretreatment with 20 μM ADR for 24 h following by cytarabine reached to a remarkable therapeutic effect (Figure 6D), indicating that ADR decreases drug resistance of MV4-11 cells through inhibition of FLT3 signal pathway.

Apart from the role in multidrug resistance, FLT3 also mediates pro-survival physiological processes that favor cancer growth and progression. In particular, two of the FLT3 downstream signaling pathways, PI3K/Akt and Ras/MAPK, are involved in proteins synthesis [46,47]. Increased protein synthesis allows for uncontrolled growth and proliferation of cancer cells as well as selective translation of specific regulatory proteins that promotes cancer progression and confers resistance to therapeutic regimens. Notably, proteomic analysis revealed that ADR is involved in protein synthesis in MV4-11 cells, and affects two of the main proteins synthesis regulation pathways, EIF2 signaling and p70S6K signaling. Therefore, we hypothesized that ADR inhibits protein synthesis to suppress MV4-11 cell proliferation. In order to validate the hypothesis, we performed a Click-iT^®^ nascent protein synthesis quantification assay to detect the incorporations of the artificial amino acid azidohomoalanine (AHA), a methionine analogue that is incorporated into newly synthesized proteins and allows the dynamic monitoring of de novo protein synthesis [48,49], so as to examine the changes in the levels of protein synthesis in ADR-treated MV4-11 cells. The MV4-11 cells were treated with 43 μM ADR or CHX (an inhibitor of protein biosynthesis as a positive control) for 12 h (instead of 72 h) to ensure that the observed reduction in nascent protein synthesis was mainly attributed to diminished upstream stimulation rather than general decrease in anabolic processes during cell death. Results showed that ADR treatment significantly reduced AHA-incorporation (Figure 6F), indicating ADR inhibits proteins synthesis in MV4-11 cells. To sum up, ADR blocks FLT3 signal pathway to reduce drug resistance of MV4-11 cells and inhibits its downstream protein synthesis pathway to suppress MV4-11 cells proliferation.

### 2.6. ADR Inhibits Fatty Acid Synthesis, Cellular Iron Pool and FLT3 Signaling in NB4 Cells

**Figure 7:**
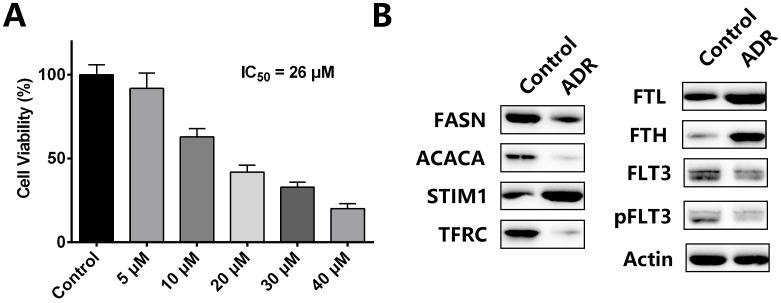
(**A**) Cell viability of NB4 cells treated with different concentration of ADR for 72 h; (**B**) the effect of ADR on the expression of proteins involve in major pathways of the study in NB4 cells.

To investigate whether ADR affects the pathways mentioned above in other AML cell lines, NB4 acute promyelocytic leukemia cell line was introduced in the study [50]. A CCK-8 assay was performed to examine the cell viability of NB4 cells treated with different concentrations of ADR for 72 h. Results showed that ADR inhibited NB4 cell proliferation in a concentration-dependent manner, and the IC50 value of ADR was 26 μM (Figure 7A). 

Therefore, we adopted 26 μM as our experimental concentration in subsequent assays. Results from western blotting showed that ADR significantly down-regulated FASN and ACACA, while up-regulated STIM1 (Figure 7B), indicating that ADR inhibits fatty acid synthesis in NB4 cells. In addition, the effect of ADR on iron pool of NB4 cells was tested with western blotting. ADR significantly down-regulated TFRC, while up-regulated FTL and FTH (Figure 7B), suggesting that ADR decreases intracellular iron pool in NB4 cells through inhibition of iron uptake and retention of iron in storage repositories. In the case of FLT3 signaling, as NB4 cell line is a FLT3-wildtype cell line, ADR only suppressed FLT3 signal pathway to some extent in NB4 cells, which differed from the significant inhibitory effect in MV4-11 cells (Figure 7B). To conclude, ADR also suppresses fatty acid synthesis, cellular iron pool and FLT3 signaling in NB4 cells, indicating that the inhibitory actions of ADR on the pathways in the study also work in other AML cell lines.

## 3. Discussion

In recent years, many phytochemicals found in dietary and medicinal plants in the tropical and sub-tropical regions have been widely investigated for their potential anti-neoplastic properties [51,52]. *Andrographis paniculata* is a herb indigenous to Southeast Asian countries like China and India [53]. It has been reported that the extracts of the whole plant of *Andrographis paniculata* exhibit a variety of bioactivities, such as anticancer [54], anti-inflammatory [55], anti-allergic [56], immunostimulatory [57], antiviral [58] and hypotensive activities [59]. Notably, andrographolide (ADR), the main active component of *Andrographis paniculata*, also displays anticancer potential against various cancer cell lines, including leukemic [54], breast cancer [27], colon cancer [60], non-small cell lung cancer [61] and prostatic cancer [62] cell lines. Although a large amount of reports showed that ADR exerts its anticancer activity via affecting various cancer cellular processes, such as metabolism [62], metastasis [25], apoptosis [63], adhesion [64], cell cycle [28] and proliferation [60], its molecular mechanism of action (MOA) remains elusive. Here, we applied a quantitative proteomics approach to fully identify the differentially expressed proteins in ADR-treated MV4-11 cells, an acute myeloid leukemia (AML) cell line, thereby elucidating the molecular mechanism of ADR’s anticancer activity through subsequent proteomic analysis.

AML is characterized by the uncontrolled proliferation of immature hematopoietic precursors with reduced potential to differentiate into their downstream myeloid counterparts [1]. The anticancer effect of ADR on different AML cell lines has been broadly reported [65,66]. Furthermore, compared to other cancer types, leukemia cell lines showed the highest sensitivity to ADR on average [26]. In addition, ADR exhibits anticancer activity even in some multidrug cancer cell lines. For instance, Han et al. reported that ADR significantly increased the sensitivity of HCT-8/5FU multidrug resistant colon cancer cell line to conventional chemotherapy drugs [33]. Therefore, we take MV4-11 cell line, a FLT3 positive AML cell line that displays multidrug resistance, as our experimental system to investigate the MOA of ADR against AML cells as well as ADR’s anti-drug resistance activity.

**Figure 8:**
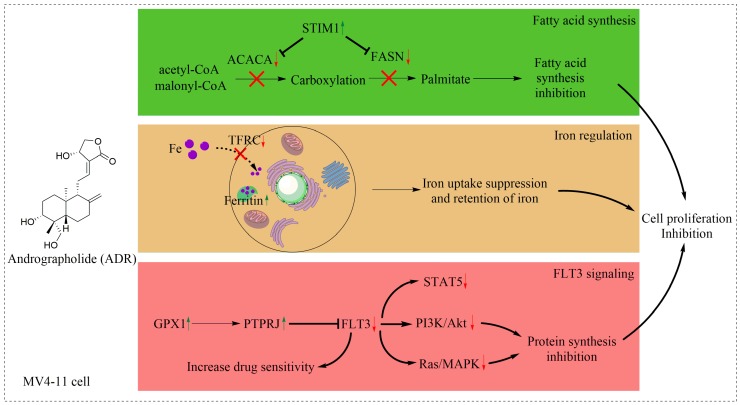
Proposed mechanism for the proliferation inhibitory effects of ADR in MV4-11 cells.

Since very few studies have been done to investigate the effect of ADR on MV4-11 cells, we started from evaluating the antiproliferation activity of ADR. Results from the cell viability assay showed that ADR inhibited MV4-11 cell proliferation in a dose- and time- dependent manner. Subsequently, a quantitative proteomics approach was applied to study the MOA of ADR on MV4-11 cells. As a result, 552 out of 3604 proteins were identified to be significantly modulated by ADR in MV4-11 cells. With proteomic analysis, we concluded that ADR inhibited MV4-11 cell proliferation through affecting three signal pathways, including inhibition of fatty acid synthesis, inhibition of iron uptake and retention of iron in storage repositories, and blockage of FLT3 signaling and its downstream protein synthesis (Figure 8).

Previous studies reported that many cancer cell types acquire fatty acid required for their metabolic demand through de novo endogenous synthesis regardless of the abundance of extracellular fatty acids [38]. Continuous lipogenesis provides cancer cells with membrane building blocks, signaling lipid molecules, posttranslational modifications of proteins as well as energy supply to support rapid cell proliferation [67]. Therefore, cancer cells often upregulate the expression and activity of enzymes that mediate the process of fatty acid synthesis at the early stage of tumorigenesis [38]. As alteration of lipid metabolism has been increasingly recognized as the prominent feature of cancer pathogenesis, inhibition of fatty acid synthesis has become a therapeutic target for cancer treatment [68].

For instance, Brusselmans et al. utilized flavonoids to inhibit fatty acid synthase activity in cancer cells to induce apoptosis [69]. Particularly, ADR’s regulatory effect on fatty acid synthesis in cancer cells has also been reported [70]. Chen et al. reported that ADR attenuated fatty acid synthase and stearoyl-CoA desaturase expression and lipid accumulation in 3T3-L1 cells [71]. In the present study, FASN and ACACA were identified to be down-regulated by ADR, suggesting that ADR inhibits fatty acid synthesis in MV4-11 cells. Schrijver et al. showed that the levels of FASN and ACACA were abnormally elevated in many human carcinomas and preneoplastic lesions, and the two proteins were closely related to aggressive cancer phenotypes and poor prognosis, revealing the therapeutic potential of the inhibition of the two proteins [72]. Furthermore, a recent study by Pardee et al. showed that the viability of HL60, Jurkat and K562 leukemia cells is greatly reduced upon inhibition of fatty acid synthesis with FASN inhibitor orlistat or ACACA inhibitor 5-(tetradecyloxy)-2-furoic acid [73], reinforcing the role of FASN and ACACA in lipid metabolism and cell proliferation. Moreover, in the present study, the addition of palmitate rescued MV4-11 cells from ADR’s cytotoxicity. Collectively, ADR inhibits FASN and ACACA expression to suppress fatty acid synthesis in MV4-11 cells, thereby inhibiting cell proliferation.

Despite similar qualitative requirement as normal cells, tumor cells often manipulate the homeostatic regulation of iron metabolism towards enhanced iron absorption and cellular uptake in order to drive rapid growth and proliferation. Therefore, many researchers consider suppression of iron uptake as a therapeutic target for cancer treatment [74]. For example, Graziadei et al. discovered that the acute-phase protein alpha 1-antitrypsin inhibited human early erythroid progenitor cell growth and proliferation through interfering with transferrin iron uptake [75]. Our proteomic data showed that ADR up-regulated Ferritin while down-regulated TFRC in MV4-11 cells. Yang et al. demonstrated that inhibition of TFRC1 expression with antisense oligonucleotide depletes intracellular iron pool required for DNA synthesis and energy metabolism, resulting in reduction of breast cancer cell viability and proliferation [76]. This suggests that ADR induced cytotoxicity and growth inhibition in MV4-11 cells may be in part due to the limitation of iron uptake by down-regulation of TFRC1. Ferritin (FT) is a ubiquitous intracellular protein which assembles into a hollow cage-like structure for storage of excess Fe^3+^ ions in the form of ferric oxyhydroxide phosphate. In a recent study, Wu et al. observed down-regulation of FT in highly tumorigenic c-myc-transformed B cells and suggested it as a transcriptional and translational effort to maximize the iron pool readily available for proliferative processes [77]. Consistently, upregulation of FT was also shown to associate with growth suppression and induction of differentiation in hematopoietic cell systems [78], suggesting that the increased expression of FT also contributes to the depletion of intracellular iron pool in MV4-11 cells. To conclude, ADR depletes intracellular iron pool through down-regulating TFRC and up-regulating FT, so as to inhibit MV4-11 cell proliferation.

Apart from the mechanism of its anticancer activity, ADR’s anti-drug resistance mechanism was also investigated. Reports showed that FLT3 signaling drives the multidrug resistance in AML cells [79,80], and many therapies used FLT3 as a target for AML treatment to decrease drug resistance [81]. Proteomic data showed that ADR down-regulated FLT3 while up-regulated PTPRJ and GPX1 in MV4-11 cells. PTPRJ, also known as density-enhanced phosphatase 1 (DEP1), is a member of the PTP superfamily which catalyzes the hydrolysis of phosphate groups from tyrosine-phosphorylated proteins [81]. It was known to negatively regulate FLT3 phosphorylation and downstream signaling activity. Depletion of PTPRJ was associated with site-selective hyperphosphorylation of FLT3 which results in enhanced FLT3-dependent activation of STAT5 and ERK1/2 as well as higher cell proliferation capacity [82]. A recent study showed that PTPRJ activity in FLT3-ITD expressing AML cells tended to be diminished compared to their WT-FLT3 expressing counterparts. In addition, activated PTPRJ was shown to inhibit cell transformation in vitro and extend survival of mice in the 32D cell/C3H/HeJ mouse model of FLT3 ITD–driven myeloproliferative disease [83]. GPX is an antioxidant enzyme involved in catalyzing the decomposition of harmful H_2_O_2_. The observed up-regulation of these antioxidant enzymes suggests a possible counteractive measure of ADR to reverse the H_2_O_2_-induced inactivation of PTPRJ, allowing PTPRJ to dephosphorylate FLT3 and inhibit its downstream signaling. Taken together, ADR inhibits FLT3 signaling pathway through up regulating GPX and PTPRJ and down regulating FLT3, so as to decrease drug resistance of MV4-11 cells.

Under normal circumstances, activated FLT3 forms complex associations with many adaptor proteins which stimulate downstream effectors in the PI3K/Akt and Ras/MAPK pathways apart from its role in drug resistance [62]. The role of the PI3K/Akt and Ras/MAPK pathways in protein synthesis induction has been extensively reviewed [46,84]. Therefore, we assumed that ADR inhibits protein synthesis through suppressing FLT3 signaling in MV4-11 cells. Previous studies showed that an increase in protein synthesis could lead to enhanced cell growth and proliferation [85]. In addition, increased protein synthesis can also result in selective translation of specific regulatory proteins that promote cancer progression and confer resistance to therapeutic regimens. Hence, the inhibition of protein synthesis has become an effective target for cancer therapy [86]. For instance, SJ Martin et al. reported that the inhibition of protein synthesis led to induction of apoptosis in human leukemic HL60 cells [87]. With a Click-iT^®^ nascent protein synthesis quantification assay, we validated the hypothesis that ADR inhibits protein synthesis to suppress cell proliferation in MV4-11 cells. Therefore, our study reinforces the anticancer activity of ADR and elucidates the mechanism of ADR’s cytotoxicity and anti-drug resistance on MV4-11 cells.

## 4. Materials and Methods

### 4.1. Cell Culture

MV4-11 (CRL-9591^TM^) immortal AML cell line was obtained from the American Type Culture Collection^®^ (Manassas, VA, USA). The blast cells were cultured in standard T-75 flasks in a humidified incubator at 37 °C with 5% CO_2_. RPMI-1640 (Sigma-Aldrich^®^, St. Louis, MO, USA) supplemented with 10% fetal bovine serum (FBS, Gibco^®^, Invitrogen, Eugene, OR, USA), 1% streptomycin and penicillin (Gibco^®^) and 2.2 g/L sodium bicarbonate was used. Aseptic conditions were strictly maintained and the cells were subcultured at a ratio of 1:5 every 2–3 days when 70–80% confluency is reached. NB4 (BNCC337678) acute promyelocytic leukemia cell line was obtained from the China Center for Type Culture Collection (CCTCC, Wuhan, China). The cells were cultured in a humidified incubator at 37 °C with 5% CO_2_. RPMI-1640 (Sigma-Aldrich^®^) supplemented with 10% inactivated Fetal Bovine Serum (FBS, Gibco^®^), and 1% streptomycin and penicillin (Gibco^®^).

### 4.2. Antibodies

Antibodies used in the study were purchased form Abcam (Cambridge, UK) and Cell Signaling Technology (Danvers, MA, USA): actin (CST, #3700); SULT1A2 (Abcam, ab123976); PRSS1 (Abcam, ab200996); INTS4 (Abcam, ab75253); PFDN1 (Abcam, ab151708); CIAPIN1 (Abcam, ab154904); FASN (CST, #3180); ACACA (CST, #3662); STIM1 (Abcam, ab57834); TFRC (Abcam, ab84036); FTL (Abcam, ab218400); FTH (Abcam, ab77127); FLT3 (CST, #3462); pFLT3 (CST, #4577); STAT5 (CST, #25656); pSTAT5 (CST, #4322); PTPRJ (Abcam, ab181244); GPX1 (CST, #3206).

### 4.3. CCK-8 Cell Viability Assay

5 × 10^3^ MV4-11 or NB4 cells were cultured in 96-well plate overnight at 37 °C. Then the cells were incubated with different concentrations of ADR for 72 h, and followed by the addition of 10 μL CCK-8 reagent per well and incubation for 4 h at 37 °C. Absorbance at 650 nm was measured with a microplate reader.

### 4.4. iTRAQ Labelling

Cells were first lysed and proteins were extracted. Tris-(2-carboxyethyl) phosphine (TCEP) was added into 100 µg of proteins from cell lysate, and followed by cysteine blocking with methyl methane-thiosulfonate (MMTS). After dilution for 20 times, the samples were digested with trypsin at 37 °C for 16 h. For each sample, its respective iTRAQ reagent (113, 114, 115 or 116) was added and incubated for 2 h at room temperature. After iTRAQ reagent labeling, all the sampled were pooled together, purified with strong cation exchange (SCX), and desalted with Sep-Pak C18 cartridges. Finally, the samples were dried with a vacuum and re-dissolving in 5 mM KH_2_PO_4_ and 5% acetonitrile.

### 4.5. 1D LC-MS/MS Analysis

Peptides labelled with different iTRAQ reagents were separated with an Eksigent NanoLC-Ultra system (Eksigent Technologies, Dublin, CA, USA) and a cHiPLC-Nanoflex system (Thermo Scientific, Rockford, IL, USA). Peptides were separated by a gradient formed by mobile phase A (2% ACN, 0.1% FA) and mobile phase B (98% ACN, 2% H_2_O, and 0.05% FA) from 12–40% of mobile phase B in 90 min, at a flow rate of 300 nL/min. TripleTOF 5600 analyzer (SCIEX, Foster City, CA, USA) was applied for MS analysis. The MS spectra were collected covering the mass range from 350 to 1250 *m*/*z*, and the accumulation time for each spectrum was 250 ms. For each mass spectrum, a maximum of 20 precursors with a charge state between +2 and +4 were picked out for fragmentation. Moreover, for each mass spectrum, signals were accumulated for 100 ms and dynamic exclusion for 15 s. High sensitivity mode was selected to measure the spectra.

### 4.6. Peptide and Protein Identification, Data Analysis

Peptides were quantified and identified with ProteinPilot Software (4.5, Applied Biosystems) overall. In the present study, two biological replicates were applied to reach a confident result. Student’s *t* test was employed to evaluate the significance of the ADR-regulated proteins, and only proteins with the *p* value < 0.05 were picked out for subsequent analysis. Strict thresholds were selected to distinguish up-regulated proteins and down-regulated proteins. 

### 4.7. Western Blotting Assay

The MV4-11 cells or NB4 cells were first treated, harvested and lysed. Proteins from cell lysate were separated with 10% SDS-PAGE gel and then transferred to a nitrocellulose membrane (HATF00010, Millipore, Billerica, MA, USA). The membrane transfer was conducted in a cold room, on ice for 90 min with a constant current of 300 mA. The membrane was then blocked with 5% non-fat milk in PBST for 1 h at room temperature with gentle shaking. Subsequently, the membrane was washed 3 times with PBST and incubated overnight with the primary antibody. Following successive washing with PBST, the membrane was incubated with the corresponding secondary antibody for 1 h at room temperature. After washing, proteins were detected with an ECL detection reagent (34075, Thermo Scientific, Rockford, IL, USA).

### 4.8. GC/MS Analysis for Fatty Acid Content

Sample was prepared according to previous publications [88,89]. Cells were lysed and dried in a nitrogen atmosphere, and fatty acid within the lysate was transesterified. Then GC/MS was applied to analyze the fatty acid methyl esters. Therefore, the average fatty acids content equals the number of double bonds multiplying the percentage.

### 4.9. Nascent Protein Synthesis Quantification Using Click Chemistry

The Click-iT^®^ Metabolic Labeling Reagents for Proteins and Click-iT^®^ Cell Reaction Buffer Kit (Molecular Probes^®^) were used to evaluate the effect of ADR on the nascent protein synthesis activity of MV4-11 cells. First, the cells were treated with the 43 μM ADR or 10 uM cycloheximide (CHX)—an inhibitor of protein biosynthesis. After a 12 h treatment, the cells were harvested and washed twice with 1 × PBS. The cells were depleted of their methionine reserves by incubating them in methionine-free RPMI for 45 min. Next, 50 µM of Click-iT^®^ AHA (l-azidohomoalanine) was added to the methionine-free growth medium and the cells were incubated for another 4 h. The cells were then harvested and fixed in 4% paraformaldehyde (PFA) for 15 min. This was followed by permeabilisation with 0.1% Triton-X 100 for 15 min. After washing the cells with 3% BSA, 0.5 mL of the Click-iT^®^ Reaction cocktail was added to each sample and the mixture was incubated for 2 h at room temperature. The cells were washed once with 3% BSA and the fluorescence was measured using a BD FACS Calibur flow cytometer (BD Biosciences, Franklin Lakes, NJ, USA), set to capture the fluorescence of Alexa Fluor 488 dye.

### 4.10. Statistical Analysis

Data in the study was analyzed with the software of GraphPad prism (5.0, GraphPad Software, La Jolla, CA, USA). Student’s t test was employed to evaluate the significant differences between groups. Only the results with *p* value < 0.05 were considered to be significant.

## 5. Conclusions

With a cell viability assay, we first discovered that ADR inhibited MV4-11 cell proliferation in a dose- and time-dependent manner. To elucidate the mechanism involved, we applied a quantitative proteomics approach to identify differentially expressed proteins in ADR-treated MV4-11 cells. As a result, 552 proteins involved in a variety of cellular processes and signaling pathways were identified to be significantly regulated by ADR. With subsequent assays, we validated that ADR inhibits fatty acid synthesis, suppresses iron uptake and induces retention of iron in storage repositories, and blocks FLT3 signaling in MV4-11 cells. Therefore, we conclude that ADR suppresses MV4-11 cell proliferation through inhibiting fatty acid synthesis, decreasing the intracellular iron pool and inhibiting protein synthesis. Furthermore, as FLT3 signaling drives drug resistance in MV4-11 cells, we demonstrated that ADR reduces drug resistance in MV4-11 cells through blocking the FLT3 signaling pathway.

## Supplementary Materials

Supplementary materials are available online. Table S1: List of top 100 overexpressed proteins at 72 h post-ADR treatment, Table S2: List of top 100 underexpressed proteins at 72 h post-ADR treatment.
