# Supplementary material for: Andrographolide Suppresses MV4-11 Cell Proliferation through the Inhibition of FLT3 Signaling, Fatty Acid Synthesis and Cellular Iron Uptake
Source: Molecules. 2017 Aug 31;22(9):1444. doi: 10.3390/molecules22091444 (PMC6151431; doi:10.3390/molecules22091444)
Supplement: Supplementary file 1 [file molecules-22-01444-s001.pdf]

Supplementary Material - Andrographolide suppresses MV4-11 cell proliferation through the inhibition of fatty acid synthesis, cellular iron uptake and FLT3 signaling

Table S1 List of top 100 overexpressed proteins at 72h post-ADR treatment.

No.	Accession	Name	Mean Ratio	Mean FC
1	IPI00300027	SULT1A2 Sulfotransferase 1A2	2.7711	1.4211
2	IPI00945846	PRSS1 Uncharacterized protein	2.7621	1.3353
3	IPI00446765	INTS4 Isoform 1 of Integrator complex subunit 4	2.7097	1.4272
4	IPI00022314	SOD2 Superoxide dismutase [Mn], mitochondrial	2.6339	1.3956
5	IPI00619898	NQO1 NQO1 protein (Fragment)	2.5569	1.3522
6	IPI00004669	GALNT2 Polypeptide N-acetylgalactosaminyltransferase 2	2.5128	1.2200
7	IPI00550948	TNFAIP8L2 Tumor necrosis factor alpha-induced protein 8-like protein 2	2.4364	1.2048
8	IPI00169285	PLBD2 Putative phospholipase B-like 2	2.4309	1.2789
9	IPI00815995	MKLN1 muskelin isoform 1	2.2551	1.1335
10	IPI00398779	PLEC Isoform 4 of Plectin	2.1669	1.0967
11	IPI00003856	ATP6V1E1 V-type proton ATPase subunit E 1	2.1578	1.1071
12	IPI00012585	HEXB Beta-hexosaminidase subunit beta	2.0910	1.0612
13	IPI01012504	PGD 6-phosphogluconate dehydrogenase, decarboxylating	2.0046	1.0030
14	IPI00011268	RALY cDNA FLJ77422, highly similar to Homo sapiens RNA binding protein, autoantigenic (hnRNP-associated with lethal yellow homolog)	1.9629	0.9360
15	IPI00006592	MSRA Isoform 1 of Mitochondrial peptide methionine sulfoxide reductase	1.9397	0.9544
16	IPI00007778	CTBS Di-N-acetylchitobiase	1.9145	0.9366
17	IPI01014563	FTL Ferritin light chain	1.9126	0.9332
18	IPI00339361	TLK1 Isoform 1 of Serine/threonine-protein kinase tousled-like 1	1.9073	0.8849
19	IPI00410714	HBA2;HBA1 Hemoglobin subunit alpha	1.9003	0.9067
20	IPI00647720	SRRM1 Isoform 1 of Serine/arginine repetitive matrix protein 1	1.8790	0.9020
21	IPI00299413	GABPA GA-binding protein alpha chain	1.8446	0.8635
22	IPI00873020	PSAP Uncharacterized protein	1.8316	0.8719
23	IPI00032313	S100A4 Protein S100-A4	1.8311	0.8492
24	IPI00644855	BROX Uncharacterized protein	1.8275	0.8298
25	IPI00010882	DFFA Isoform DFF45 of DNA fragmentation factor subunit alpha	1.8242	0.8627
26	IPI00301163	POGLUT1 Protein O-glucosyltransferase 1	1.8046	0.8251
27	IPI00946775	AKAP2 Uncharacterized protein	1.7288	0.7609
28	IPI01013419	FTH Ferritin heavy chain	1.7132	0.7749
29	IPI00013968	COX7C Cytochrome c oxidase subunit 7C, mitochondrial	1.7198	0.7701
30	IPI00967902	C6orf108 26 kDa protein	1.6947	0.7278
31	IPI00980505	CCS 27 kDa protein	1.6916	0.7528
32	IPI00657860	BST1 Uncharacterized protein	1.6885	0.7537
33	IPI00473011	HBD Hemoglobin subunit delta	1.6874	0.7542
34	IPI00010180	CES1 Isoform 1 of Liver carboxylesterase 1	1.6804	0.7484
35	IPI00061229	OMA1 Isoform 1 of Metalloendopeptidase OMA1, mitochondrial	1.6695	0.7234
36	IPI00299024	BASP1 Isoform 1 of Brain acid soluble protein 1	1.6683	0.7360

37	IPI00030059	GNG10 Guanine nucleotide-binding protein G(I)/G(S)/G(O) subunit gamma-10	1.6630	0.7328
38	IPI00847689	HTATIP2 Isoform 3 of Oxidoreductase HTATIP2	1.6539	0.7192
39	IPI00909791	TBXAS1 thromboxane-A synthase isoform 3	1.6420	0.6946
40	IPI00470674	CYB5R1 NADH-cytochrome b5 reductase 1	1.6416	0.6952
41	IPI00329054	OSTM1 Osteopetrosis-associated transmembrane protein 1	1.6371	0.6744
42	IPI00217766	SCARB2 Lysosome membrane protein 2	1.6349	0.7031
43	IPI00021983	NCSTN Isoform 1 of Nicastrin	1.6321	0.7039
44	IPI00974532	TOR1AIP1 68 kDa protein	1.6114	0.6846
45	IPI00412399	TRPM2 Isoform 2 of Transient receptor potential cation channel subfamily M member 2	1.6105	0.6825
46	IPI00009634	SQRDL Sulfide:quinone oxidoreductase, mitochondrial	1.6058	0.6820
47	IPI00414928	IL4I1 Isoform 2 of L-amino-acid oxidase	1.5889	0.6642
48	IPI00304925	HSPA1B;HSPA1A Heat shock 70 kDa protein 1A/1B	1.5868	0.6655
49	IPI00019373	TMEM184C Isoform 1 of Transmembrane protein 184C	1.5820	0.6300
50	IPI00477619	C19orf2 Isoform 1 of Unconventional prefoldin RPB5 interactor	1.5794	0.6413
51	IPI00171856	DOHH Deoxyhypusine hydroxylase	1.5774	0.6238
52	IPI00009268	ACY1;ABHD14A cDNA FLJ60317, highly similar to Aminoacylase-1	1.5772	0.6403
53	IPI00000861	LASP1 Isoform 1 of LIM and SH3 domain protein 1	1.5758	0.6238
54	IPI00376035	C6orf120 cDNA FLJ55847	1.5720	0.6525
55	IPI00031064	TMEM126A Transmembrane protein 126A	1.5719	0.6469
56	IPI00007955	TRIM16 Isoform 1 of Tripartite motif-containing protein 16	1.5694	0.6343
57	IPI00925213	MTX2 Uncharacterized protein	1.5678	0.6423
58	IPI00921805	EVI2B cDNA FLJ55224, highly similar to EVI2B protein	1.5670	0.6341
59	IPI00938010	ATRX Isoform 2 of Transcriptional regulator ATRX	1.5594	0.6266
60	IPI00032003	EMD Emerin	1.5523	0.6291
61	IPI00743576	ATP6V0A1 Isoform 2 of V-type proton ATPase 116 kDa subunit a	1.5499	0.6021
62	IPI00009896	EPHX1 Epoxide hydrolase 1	1.5467	0.6286
63	IPI00001541	TIMM9 Mitochondrial import inner membrane translocase subunit Tim9	1.5445	0.6154
64	IPI00477441	LAMTOR2 Isoform 2 of Ragulator complex protein LAMTOR2	1.5399	0.6197
65	IPI00164150	RAB44 Ras-related protein Rab-44	1.5395	0.6163
66	IPI00028383	FAM173A Protein FAM173A	1.5297	0.6008
67	IPI01019118	NDRG1 cDNA FLJ39243 fis, clone OCBBF2008283, highly similar to Protein NDRG1	1.5292	0.6042
68	IPI00884105	LAMP1 Lysosome-associated membrane glycoprotein 1	1.5280	0.6042
69	IPI00782974	C17orf85 Isoform 1 of Uncharacterized protein C17orf85	1.5262	0.6100
70	IPI00306400	KIFC1 Kinesin-like protein KIFC1	1.5189	0.5822
71	IPI00001699	PYCARD Isoform 1 of Apoptosis-associated speck-like protein containing a CARD	1.5174	0.5936
72	IPI00921897	BLM cDNA FLJ56969, highly similar to Bloom syndrome protein	1.5122	0.5856
73	IPI00001545	WAS Wiskott-Aldrich syndrome protein	1.5104	0.5882
74	IPI00412499	RABEP2 Isoform 1 of Rab GTPase-binding effector protein 2	1.5091	0.5743

75	IPI00940936	LYN LYN protein (Fragment)	1.5029	0.5874
76	IPI01025991	ZC3H14 80 kDa protein	1.4983	0.5694
77	IPI00745343	LOC100130932 Small nuclear ribonucleoprotein G-like protein	1.4961	0.5754
78	IPI00853163	TYMP Uncharacterized protein	1.4959	0.5732
79	IPI00298971	VTN Vitronectin	1.4926	0.5759
80	IPI00005055	CXorf56 UPF0428 protein CXorf56	1.4926	0.5667
81	IPI00328840	THOC4 THO complex subunit 4	1.4894	0.5741
82	IPI01014285	PITPNB Isoform 2 of Phosphatidylinositol transfer protein beta isoform	1.4871	0.5441
83	IPI00021405	LMNA Isoform A of Prelamin-A/C	1.4866	0.5693
84	IPI00032831	SNAP29 Synaptosomal-associated protein 29	1.4864	0.5667
85	IPI00253036	CD99 Isoform I of CD99 antigen	1.4864	0.5616
86	IPI00220967	RRBP1 Isoform 1 of Ribosome-binding protein 1	1.4843	0.5697
87	IPI00386947	ARHGAP17 Isoform 2 of Rho GTPase-activating protein 17	1.4836	0.5647
88	IPI00641157	CTSA lysosomal protective protein isoform c precursor	1.4818	0.5673
89	IPI00303071	CECR1 Isoform 1 of Adenosine deaminase CECR1	1.4807	0.5396
90	IPI00939644	CCNK Uncharacterized protein	1.4807	0.5540
91	IPI00219673	GSTK1 Isoform 1 of Glutathione S-transferase kappa 1	1.4796	0.5647
92	IPI00013698	ASAH1 N-acylsphingosine amidohydrolase (Acid ceramidase) 1	1.4759	0.5482
93	IPI00409717	MIR1248;SNORA4;SNORD2;EIF4A2;SNORA81;SNORA63 Isoform 2 of Eukaryotic initiation factor 4A-II	1.4744	0.5572
94	IPI00843910	FUCA1 Tissue alpha-L-fucosidase	1.4741	0.5488
95	IPI00215999	CDK11B Isoform SV9 of Cyclin-dependent kinase 11B	1.4721	0.5446
96	IPI00439194	MBD3 Isoform 1 of Methyl-CpG-binding domain protein 3	1.4712	0.5550
97	IPI00011635	BCL2L13 Isoform 2 of Bcl-2-like protein 13	1.4617	0.5458
98	IPI00479912	PXK Isoform 1 of PX domain-containing protein kinase-like protein	1.4616	0.5219
99	IPI00016457	CRAT Isoform 1 of Carnitine O-acetyltransferase	1.4546	0.5257
100	IPI00783726	KTN1 kinectin isoform b	1.4533	0.5391

Table S2 List of top 100 underexpressed proteins at 72h post-ADR treatment.

No.	Accession	Name	Mean Ratio	Mean FC
1	IPI00872143	MT1F Metallothionein	0.4160	-1.2714
2	IPI00000051	PFDN1 Prefoldin subunit 1	0.4174	-1.3799
3	IPI00387130	CIAPIN1 Isoform 1 of Anamorsin	0.4188	-1.3215
4	IPI00028064	CTSG Cathepsin G	0.4498	-1.1638
5	IPI00183786	FADS2 Isoform 1 of Fatty acid desaturase 2	0.4537	-1.1486
6	IPI00305552	CHCHD5 cDNA FLJ39671 fis, clone SMINT2008917	0.4670	-1.1702
7	IPI00301434	BOLA2B;BOLA2 BolA-like protein 2	0.4819	-1.1403
8	IPI00514622	RANBP6 Ran-binding protein 6	0.4898	-1.0319
9	IPI00020226	ACOX3 Isoform 1 of Peroxisomal acyl-coenzyme A oxidase 3	0.4923	-1.0478
10	IPI00028481	RAB8A Ras-related protein Rab-8A	0.4961	-1.0460
11	IPI00925572	ASNS asparagine synthetase [glutamine-hydrolyzing] isoform b	0.4982	-1.0500
12	IPI00922479	REVERSED PEX14 Isoform 2 of Peroxisomal membrane protein PEX14	0.4989	-1.0065
13	IPI00745921	RNF126 Isoform 1 of RING finger protein 126	0.5060	-1.0247
14	IPI00185146	IPO9 Importin-9	0.5060	-0.9871
15	IPI00026850	TSPO Translocator protein	0.5163	-0.9752
16	IPI00103940	MFSD10 Major facilitator superfamily domain-containing protein 10	0.5209	-0.9418
17	IPI00981027	PCYT2 ethanolamine-phosphate cytidylyltransferase isoform 1	0.5228	-0.9612
18	IPI00219049	TGOLN2 Isoform TGN48 of Trans-Golgi network integral membrane protein 2	0.5320	-0.9548
19	IPI01025455	DCAF8 cDNA FLJ55296, highly similar to Homo sapiens WD repeat domain 42A (WDR42A), mRNA	0.5323	-0.9350
20	IPI00012795	EIF3I Eukaryotic translation initiation factor 3 subunit I	0.5410	-0.9087
21	IPI00302860	DHX33 cDNA FLJ56443, highly similar to Putative ATP-dependent RNA helicase DHX33	0.5493	-0.9355
22	IPI00925334	ARPC4;ARPC4-TTLL3 Uncharacterized protein	0.5546	-0.8595
23	IPI00300620	IFITM1 Interferon-induced transmembrane protein 1	0.5561	-0.8561
24	IPI00031804	VDAC3 Isoform 1 of Voltage-dependent anion-selective channel protein 3	0.5565	-0.8513
25	IPI00006408	NOSIP Nitric oxide synthase-interacting protein	0.5583	-0.8846
26	IPI00183666	TRPV2 Transient receptor potential cation channel subfamily V	0.5590	-0.8551
27	IPI00022597	UBE2M NEDD8-conjugating enzyme Ubc12	0.5641	-0.8519
28	IPI00239815	CIRH1A Isoform 1 of Cirhin	0.5653	-0.8299
29	IPI00006658	PIN4 Isoform 2 of Peptidyl-prolyl cis-trans isomerase NIMA-interacting 4	0.5662	-0.8701
30	IPI00917650	PIEZO1 Protein PIEZO1	0.5673	-0.8827
31	IPI00291007	SCARB1 Isoform 3 of Scavenger receptor class B member 1	0.5691	-0.8187
32	IPI00980912	MT-ND1 NADH-ubiquinone oxidoreductase chain 1	0.5704	-0.8242
33	IPI00293963	CDYL Isoform 1 of Chromodomain Y-like protein	0.5713	-0.8410
34	IPI00145593	NOM1 Nucleolar MIF4G domain-containing protein 1	0.5723	-0.8128

35	IPI00908880	APBB1IP cDNA FLJ61520, highly similar to Amyloid beta A4 protein-bindingfamily B member 1- interacting protein	0.5904	-0.7835
36	IPI00641181	MARCKSL1 MARCKS-related protein	0.5917	-0.7692
37	IPI01009057	SSR4 translocon-associated protein subunit delta isoform 1 precursor	0.5917	-0.7614
38	IPI00654820	MT-ATP6 ATP synthase subunit a	0.5926	-0.7562
39	IPI00297492	STT3A Dolichyl-diphosphooligosaccharide--protein glycosyltransferase subunit STT3A	0.5962	-0.7592
40	IPI00181728	BRIX1 Ribosome biogenesis protein BRX1 homolog	0.5965	-0.7552
41	IPI00022462	TFRC Transferrin receptor protein 1	0.5973	-0.7471
42	IPI00178750	FAM192BP;FAM192A Protein FAM192A	0.6003	-0.7424
43	IPI00005722	FLT3 Uncharacterized protein	0.6018	-0.7331
44	IPI00031424	PI4KA Isoform 2 of Phosphatidylinositol 4-kinase alpha	0.6031	-0.7557
45	IPI00642862	PPIL4 Peptidyl-prolyl cis-trans isomerase-like 4	0.6037	-0.7432
46	IPI00878135	POLDIP3 Uncharacterized protein	0.6122	-0.7421
47	IPI00012462	EIF2A Eukaryotic translation initiation factor 2A	0.6127	-0.7081
48	IPI00304875	HIRIP3 Isoform 1 of HIRA-interacting protein 3	0.6141	-0.7607
49	IPI00916480	PTMA;MIR1244-3;MIR1244-2;MIR1244-1 Uncharacterized protein	0.6145	-0.7177
50	IPI00021167	PRKRA Isoform 1 of Interferon-inducible double stranded RNA-dependent protein kinase activator A	0.6194	-0.6975
51	IPI00395627	CACYBP Isoform 1 of Calcyclin-binding protein	0.6203	-0.6897
52	IPI00746642	TMEM48 Isoform 2 of Nucleoporin NDC1	0.6227	-0.7071
53	IPI00299084	TMEM33 Transmembrane protein 33	0.6227	-0.6871
54	IPI00025491	EIF4A1 Eukaryotic initiation factor 4A-I	0.6242	-0.6902
55	IPI00025512	HSPB1 Heat shock protein beta-1	0.6247	-0.6930
56	IPI00008569	YKT6 Synaptobrevin homolog YKT6	0.6260	-0.7003
57	IPI00033516	TUBGCP3 Isoform 1 of Gamma-tubulin complex component 3	0.6300	-0.6685
58	IPI00007166	IER3IP1 Immediate early response 3-interacting protein 1	0.6301	-0.6671
59	IPI00796462	RANP1;RAN 27 kDa protein	0.6326	-0.6612
60	IPI00472047	SIRT2 Isoform 3 of NAD-dependent deacetylase sirtuin-2	0.6331	-0.6636
61	IPI00909867	ANKRD17 cDNA FLJ57343, highly similar to Homo sapiens ankyrin repeat domain 17 (ANKRD17), transcript variant 1, mRNA (Fragment)	0.6332	-0.6936
62	IPI00216691	PFN1 Profilin-1	0.6334	-0.6606
63	IPI00008998	PTPLAD1 3-hydroxyacyl-CoA dehydratase 3	0.6346	-0.6646
64	IPI00217240	WDR75 WD repeat-containing protein 75	0.6365	-0.6589
65	IPI00305242	CASP8 Isoform 4 of Caspase-8	0.6385	-0.6753
66	IPI00106966	TMEM70 Isoform 1 of Transmembrane protein 70, mitochondrial	0.6385	-0.6808
67	IPI00815732	PAICS Isoform 2 of Multifunctional protein ADE2	0.6390	-0.6469
68	IPI00940120	RCSD1 Uncharacterized protein	0.6396	-0.6600
69	IPI00020887	VAMP7 Isoform 1 of Vesicle-associated membrane protein 7	0.6418	-0.6866
70	IPI00026202	RPL18A 60S ribosomal protein L18a	0.6446	-0.6432
71	IPI00473014	DSTN Destrin	0.6457	-0.6435
72	IPI00063408	DHTKD1 Probable 2-oxoglutarate dehydrogenase E1 component DHKTD1, mitochondrial	0.6460	-0.6563

73	IPI00019407	NSDHL Sterol-4-alpha-carboxylate 3-dehydrogenase, decarboxylating	0.6479	-0.6664
74	IPI00220528	SNRPF Small nuclear ribonucleoprotein F	0.6479	-0.6479
75	IPI00215790	RPL38 60S ribosomal protein L38	0.6487	-0.6257
76	IPI00748342	BECN1 Beclin-1	0.6494	-0.6461
77	IPI00965476	DBI acyl-CoA-binding protein isoform 5	0.6497	-0.6237
78	IPI00470573	ACTR2 actin-related protein 2 isoform a	0.6497	-0.6308
79	IPI00746655	ESYT1 Isoform 2 of Extended synaptotagmin-1	0.6520	-0.6172
80	IPI00011253	RPS3 40S ribosomal protein S3	0.6546	-0.6116
81	IPI00247583	RPL21;SNORD102;SNORA27;RPL21P19 60S ribosomal protein L21	0.6582	-0.6059
82	IPI00980672	LOC100505584 hypothetical protein LOC100505584	0.6584	-0.6111
83	IPI01009252	CRTAP cDNA FLJ34453 fis, clone HLUNG2002429, highly similar to Homo sapiens cartilage-associated protein	0.6596	-0.6205
84	IPI00797864	TMED2 cDNA FLJ52153, highly similar to Transmembrane emp24 domain-containing protein 2	0.6605	-0.5992
85	IPI00383680	RPN2 dolichyl-diphosphooligosaccharide--protein glycosyltransferase subunit 2 isoform 2 precursor	0.6645	-0.5899
86	IPI00221088	RPS9 40S ribosomal protein S9	0.6646	-0.5908
87	IPI00303207	ABCE1 ATP-binding cassette sub-family E member 1	0.6649	-0.5906
88	IPI00944945	TNPO3 transportin-3 isoform 2	0.6658	-0.5981
89	IPI00383960	EGFL7 Epidermal growth factor-like protein 7	0.6658	-0.5948
90	IPI00027350	PRDX2 Peroxiredoxin-2	0.6677	-0.5840
91	IPI00016513	RAB10 Ras-related protein Rab-10	0.6687	-0.5828
92	IPI00028491	AGPAT5 1-acyl-sn-glycerol-3-phosphate acyltransferase epsilon	0.6699	-0.5862
93	IPI00657752	CD81 Uncharacterized protein	0.6703	-0.5810
94	IPI00030847	TM9SF3 Transmembrane 9 superfamily member 3	0.6712	-0.5877
95	IPI00014367	NSMCE4A Isoform 1 of Non-structural maintenance of chromosomes element 4 homolog A	0.6719	-0.5845
96	IPI00909232	HNRNPC cDNA FLJ53542, highly similar to Heterogeneous nuclear ribonucleoproteins C	0.6723	-0.5860
97	IPI00021840	RPS6 40S ribosomal protein S6	0.6741	-0.5698
98	IPI00149650	PPWD1 Peptidylprolyl isomerase domain and WD repeat-containing protein 1	0.6744	-0.5744
99	IPI00915872	- Trinucleotide repeat containing 6B (Fragment)	0.6761	-0.5986
100	IPI00216308	VDAC1 Voltage-dependent anion-selective channel protein 1	0.6766	-0.5754
